# Identification of Potential Biomarkers Associated with Basal Cell Carcinoma

**DOI:** 10.1155/2020/2073690

**Published:** 2020-04-17

**Authors:** Yong Liu, Hui Liu, Queqiao Bian

**Affiliations:** Department of Dermatology & STD, The Third Central Hospital of Tianjin, Tianjin Key Laboratory of Artificial Cell, Artificial Cell Engineering Technology Research Center of Public Health Ministry, Tianjin Institute of Hepatobiliary Disease, Tianjin 300170, China

## Abstract

**Purpose:**

This work is aimed at identifying several molecular markers correlated with the diagnosis and development of basal cell carcinoma (BCC).

**Methods:**

The available microarray datasets for BCC were obtained from the Gene Expression Omnibus (GEO) database, and differentially expressed genes (DEGs) were identified between BCC and healthy controls. Afterward, the functional enrichment analysis and protein-protein interaction (PPI) network analysis of these screened DEGs were performed. An external validation for the DEG expression level was also carried out, and receiver operating characteristic curve analysis was used to evaluate the diagnostic values of DEGs.

**Result:**

In total, five microarray datasets for BCC were downloaded and 804 DEGs (414 upregulated and 390 downregulated genes) were identified. Functional enrichment analysis showed that these genes including *CYFIP2*, *HOXB5*, *EGFR*, *FOXN3*, *PTPN3*, *CDC20*, *MARCKSL1*, *FAS*, and *PTCH1* were closely correlated with the cell process and *PTCH1* played central roles in the BCC signaling pathway. Moreover, *EGFR* was a hub gene in the PPI network. The expression changes of six genes (*CYFIP2*, *HOXB5*, *FOXN3*, *PTPN3*, *MARCKSL1*, and *FAS*) were validated by an external GSE74858 dataset analysis. Finally, ROC analysis revealed that *CYFIP2*, HOXB5, *PTPN3*, *MARCKSL1*, *PTCH1*, and *CDC20* could distinguish BCC and healthy individuals.

**Conclusion:**

Nine gene signatures (*CYFIP2*, *HOXB5*, *EGFR*, *FOXN3*, *PTPN3*, *CDC20*, *MARCKSL1*, *FAS*, and *PTCH1*) may serve as promising targets for BCC detection and development.

## 1. Introduction

Basal cell carcinoma (BCC) is one of common malignant epithelial neoplasms that derive from basal cells and accounts for nearly 75% of skin cancers [1]. It was reported that the BCC incidence rates have been increasing and roughly reached 2.75 million cases around the world [2]. BCC was generally categorized into several types such as superficial, nodular, and nevoid BCC on the basis of its histological and clinical characteristics. Notably, the majority of lesions for BCC occurred on the head and neck [3]. The convincing evidence has suggested that gender, age, sunlight exposure (especially ultraviolet radiation), smoking, chemicals, and fair skin were all BCC risk factors [4, 5]. Although BCC recurrence was extremely frequent due to local tissue destruction, it rarely metastasizes or leads to death [6]. The imiquimod application, surgical excision, and radiation therapy have been unsatisfactory for BCC treatment. Therefore, it is imperative to develop promising strategies for early diagnosis and intervention of BCC.

Fortunately, the next-generation high-throughput sequencing provides fascinating opportunities for bioinformatics analysis. A growing number of studies have demonstrated that gene expression profiling could effectively screen molecular makers and predict pathogenesis for tumorigenesis in the last decades, thereby offering novel therapeutic strategies for cancer treatment [7]. Fang et al. previously found that the expression level of *EGR1* (early growth response 1) was decreased in BCC based on a microarray analysis. Furthermore, they also noted that *EGR1* might suppress epidermal proliferation via modulating *CDC25A* that is critical for cell cycle progression from G1 to S phase [8]. Heller et al. conducted a global gene expression analysis and identified BCC-associated differentially expressed gene (DEG) targets, providing several new diagnostic signatures for the susceptibility of BCC [9]. Later, Yunoki et al. identified three BCC-correlated genes including *BCL2* (B-cell lymphoma 2), *PTCH1* (Patched 1), and *SOX9* (SRY-box 9) and unique gene networks involved with tumorigenesis through a microarray analysis [10]. Additionally, Jee et al. classified BCC into squamous cell carcinoma- (SCC-) like BCC, normal-like BCC, and classical BCC subtypes by a comparative analysis of gene expression profiles from BCC, SCC, and normal skin tissues. Moreover, functional analysis revealed that hedgehog signaling pathways were possibly related to classical BCC development [11]. Although numerous investigations have extracted several BCC-related gene makers, a deeper insight into the underlying molecular mechanisms of BCC has not been completely achieved.

In this study, the eligible mRNA microarray datasets from BCC tissues were retrieved and downloaded from the National Center for Biotechnology Information Gene Expression Omnibus (GEO) database. Then, the DEGs between BCC and normal tissues were screened followed by functional enrichment analyses. The protein-protein (PPI) network was also constructed to explore underlying interactions among key genes. Finally, the expression levels of DEGs were further examined by an external dataset, and diagnostic performance of these genes were also evaluated. This work provided novel gene signatures for BCC diagnosis and promoted a deeper understanding for BCC progression.

## 2. Materials and Methods

### 2.1. Data Acquisition

The publicly accessible microarray datasets in BCC were searched and downloaded from the NCBI-GEO [12] database (https://www.ncbi.nlm.nih.gov/geo/). The keyword terms of carcinoma, basal cell (MeSH Terms) OR basal cell carcinoma (All Fields) AND “*Homo sapiens*” (porgn) AND “gse” (Filter) were used for precise searching. The inclusive criteria for datasets were as follows: (i) the datasets were gene expression profiles, (ii) the data was obtained from tumor tissues of patients with BCC or normal skin tissues from healthy individuals (normal controls), and (iii) the patients did not receive medication or other treatments. Finally, five datasets (GSE103439, GSE53462, GSE42109, GSE39612, and GSE7553) were chosen according to the abovementioned screening criteria as shown in Table 1. Of these, four datasets (GSE103439, GSE42109, GSE39612, and GSE7553) were generated by the GPL570 [HG-U133_Plus_2] Affymetrix Human Genome U133 Plus 2.0 Array platform, and the platform for GSE53462 was GPL10558 Illumina Human HT-12 V4.0 expression beadchip. These datasets would be used for the following integrated analyses, which consisted of 48 BCC tissues and 85 normal tissue samples.

### 2.2. Identification of Differentially Expressed Genes (DEGs)

A meta-analysis of five microarray datasets from different platforms was carried out according to the guidelines provided by Ramasamy et al. [13]. The preprocessing of raw data across different platforms, mainly including background correction, normalization, log_2_ transformation, and gene expression calculation, was performed. The overlapped genes in five datasets were selected. Then, the R metaMA package was used to calculate effect sizes from unpaired data by either classical or moderated *t*-tests (Limma) for each study and combine these effect sizes [14]. The DEGs between BCC and normal controls were screened according to the threshold of the multiple comparison correction false discovery rate (FDR) < 0.05. The whole R sentences for data processing and identification of DEGs in this study were shown in Supplementary Materials (available here). Finally, the hierarchical clustering analysis of identified DEGs was performed by the pheatmap package (https://cran.r-project.org/package=pheatmap) in R language.

### 2.3. Gene Ontology (GO) and Pathway Enrichment Analyses

To explore the possible biological roles of the DEGs, the functional analyses were conducted. Firstly, the GO analysis involving three categories of molecular function (MF), cellular component (CC), and biological process (BP) was conducted with BiNGO plugin in cytoscape software [15]. The parameters were set as follows: (i) hypergeometric test was used as the selected statistical test; (ii) Benjamini and Hochberg FDR correction was set as selected correction; (iii) selected significance level was 0.05; and (iv) for testing option, we used whole annotation as the reference set. In addition, the Kyoto Encyclopedia of Genes and Genomes (KEGG) pathway analysis was undertaken using KOBAS (http://kobas.cbi.pku.edu.cn/index.php), which is a web-accessible tool and could identify enriched pathways for an input set of genes by mapping to genes using known pathways in the KEGG database [16]. Moreover, the statistically significantly enriched pathways were selected with the cutoff of *P* value < 0.05.

### 2.4. Protein-Protein Interaction (PPI) Network

The Biological General Repository for Interaction Datasets (BioGRID) database is available online and provides 1,728,498 biological PPI interactions by August 2019. Here, we performed a PPI analysis for screened DEGs based on this database to identify significant protein pairs. The cytoscape software (http://apps.cytoscape.org/apps/cytonca) was utilized to construct the PPI network. Moreover, the CytoNCA [17] (http://apps.cytoscape.org/apps/cytonca), a cytoscape plugin, was used to analyze topological characteristics of PPI nodes using the without weight parameter. Several metrics such as Degree Centrality (DC), Betweenness centrality (BC), and Closeness centrality (CC) were estimated. In this study, hub protein was determined on the basis of high scores. In addition, the MCODE plugin (http://apps.cytoscape.org/apps/mcode) in cytoscape software was employed to further identify several PPI submodules using the default parameters of degree cutoff = 2, node score cutoff = 0.2, *K*‐core = 2, and max.depth = 100 [18]. Notably, the PPI score > 3 was considered as the cutoff for screening significantly enriched functional modules from the PPI network.

### 2.5. Validation of DEG Expression Levels by a Noncoding RNA Expression Profile

The GSE74858 dataset, including 3 BCC patients and 3 healthy individuals, was obtained from the GEO database and used as a verification dataset to evaluate the expression levels of DEGs. Furthermore, the receiver operating characteristic (ROC) analysis was also carried out to assess the performance of DEGs in this research using the “pROC” package (https://cran.r-project.org/web/packages/pROC/index.html) in R language. The area under the ROC curve (AUC) was then computed. Herein, if AUC values of the genes were greater than 0.9, these genes were considered to discriminate patients undergoing BCC from healthy controls with high specificity and sensitivity.

## 3. Results

### 3.1. DEG Screening

**Figure 1:**
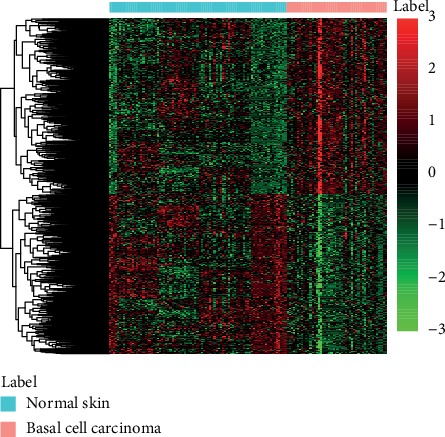
The heatmap of differentially expressed genes in basal cell carcinoma. The “label” represents sample type (basal cell carcinoma and normal skin samples). The light red color shows the basal cell carcinoma samples, and light blue color shows normal skin samples.

The meta-analysis of five gene expression profiles was conducted, and the DEGs were identified. Consequently, a total of 804 DEGs were extracted between BCC and normal controls according to screening criteria described in Materials and Methods. Of these, there were 414 upregulated genes and 390 downregulated genes in patients with BCC. Moreover, the clustering analysis of these DEGs indicated that they could clearly distinguish BCC and healthy controls as exhibited in Figure 1, suggesting that the differential gene expression was possibly responsible for BCC occurrence.

### 3.2. Functional Enrichment Analyses of DEGs

**Figure 2:**
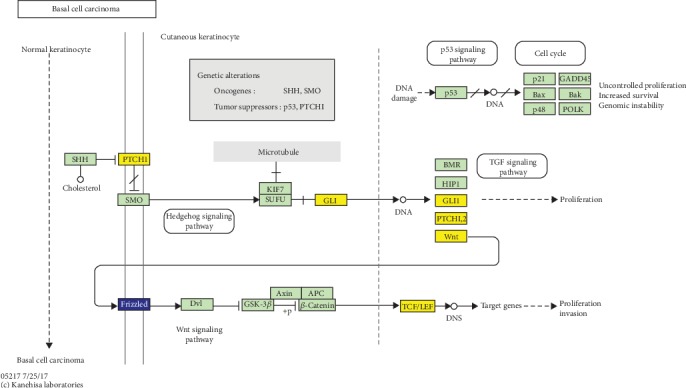
Basal cell carcinoma pathway from the Kyoto Encyclopedia of Genes and Genomes enrichment analysis. The yellow color shows the upregulated genes while blue color represents the downregulated genes.

As shown in Table 2, the GO functional annotation analysis showed that identified DEGs were enriched in 53 GO-BP terms including cellular component organization, cell cycle, cell proliferation, and ectoderm development. Meanwhile, they were focused on 54 GO-CC terms such as intracellular part, intracellular, and cytoplasm. It was also observed that there were three GO-MF terms (protein binding, binding, and ribonuclease H activity). Notably, numerous genes were closely associated with the cellular process, including *CYFIP2* (cytoplasmic FMR1 interacting protein 2; upregulated), *HOXB5* (homeobox B5; downregulated), *EGFR* (epidermal growth factor receptor; downregulated), *FOXN3* (forkhead box N3; upregulated), *PTPN3* (protein tyrosine phosphatase nonreceptor type 3; downregulated), *CDC20* (cell division cycle 20; upregulated), *MARCKSL1* (myristoylated alanine rich protein kinase C substrate like 1; upregulated), *FAS* (fas cell surface death receptor; downregulated), and upregulated *PTCH1*. More specifically, *FOXN3*, *CDC20*, and *EGFR* played essential roles in the cell cycle process, and *PTCH1* and *EGFR* were involved in cell proliferation. Additionally, KEGG pathway enrichment analysis revealed that these genes were primarily enriched in pathways in cancer, p53 signaling pathway and BCC pathway. We also noted that *PTCH1* exerted critical biological roles in the BCC pathway (Figure 2).

### 3.3. PPI Analysis

**Figure 3:**
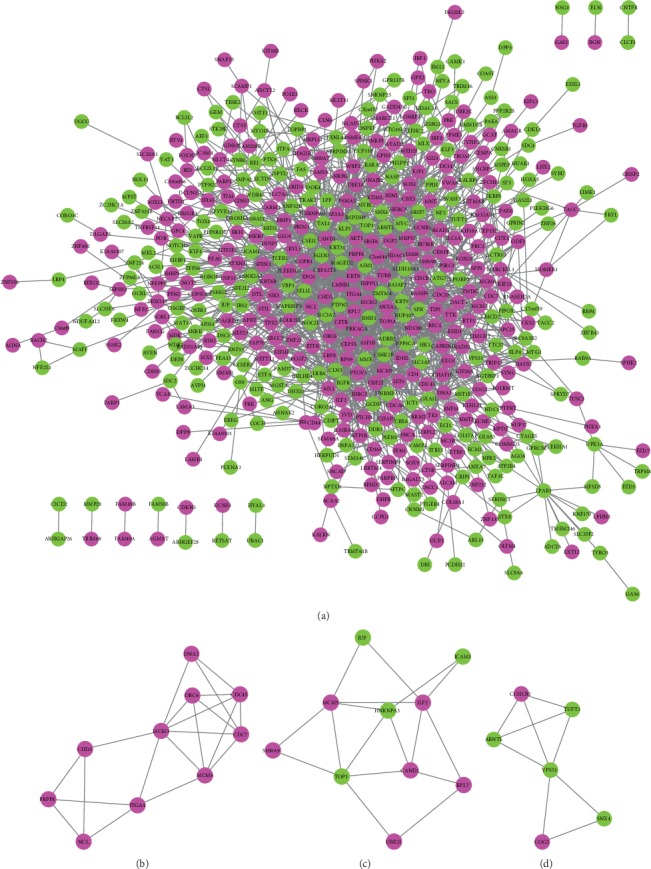
The protein-protein interaction (PPI) network analysis: (a) PPI network of differentially expressed genes: (b) submodule 1; (c) submodule 2; (d) submodule 3. The submodules in the PPI network were extracted by MCODE based on PPI score > 3. The rosy red circular nodes denote upregulated genes. The green circular nodes represent significantly downregulated genes. The node size represents degree.

PPI network analysis was conducted to explore the underlying interactions of DEGs. In total, 549 gene nodes (296 upregulated genes and 254 downregulated genes) and 1462 protein pairs were obtained in the PPI network (Figure 3(a)). The top 5 gene nodes with a high degree were *EGLN3* (Egl-9 family hypoxia inducible factor 3; degree = 82), *XPO1* (exportin 1; degree = 81), *EGFR* (degree = 77), *CFTR* (CF transmembrane conductance regulator; degree = 48), and *MCM2* (minichromosome maintenance complex component 2; degree = 47), and they were regarded as hub genes. Furthermore, the PPI submodule analysis suggested that 3 significantly enriched submodules were identified based on the PPI score > 3 (Figures 3(b)–3(d)). Interestingly, we found that the genes in submodule 1 were all upregulated.

### 3.4. Verification of DEG Expression Levels and ROC Analysis

**Figure 4:**
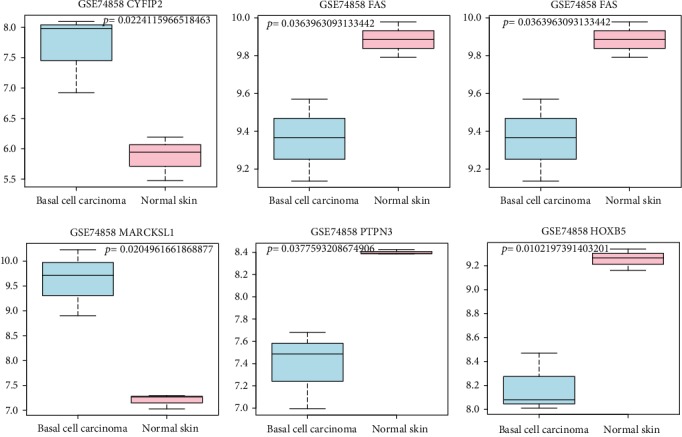
Validation of the expression levels of six differentially expressed genes in T basal cell carcinoma based on GSE74858 dataset. The *x*-axis represents basal cell carcinoma and normal controls. The *y*-axis represents the expression read counts.

**Figure 5:**
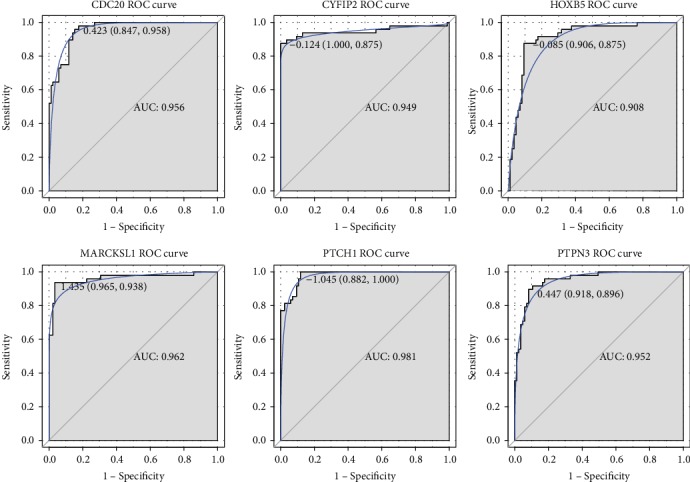
The receiver operating characteristic (ROC) curve analyzed the diagnostic value of six differentially expressed genes in basal cell carcinoma. The ROC curves were used to evaluate the diagnostic value of these six differentially expressed genes in basal cell carcinoma with sensitivity and 1-specificity. The *x*-axis shows 1-specificity, and the *y*-axis shows sensitivity. AUC: area under the ROC curve.

A noncoding RNA profile GSE74858 was downloaded from the GEO repository, and there were 145 overlapped genes between this dataset and the integrated datasets. We found that 25 overlapped genes exhibited significant differential expression, and their expression trends were also consistent with our integration analysis. More notably, six genes (*CYFIP2*, *HOXB5*, *FOXN3*, *PTPN3*, *MARCKSL1*, and *FAS*) were predominately correlated with the cellular biological process, and their expression patterns were validated by an external GSE74858 dataset (Figure 4). Besides, the ROC curve analysis was conducted to assess the diagnostic values of DEGs in BCC by five microarray datasets. There were 439 genes according to AUC > 0.9, and 129 genes were overlapped with DEGs by integrated analysis and then extracted. Our findings revealed that *CYFIP2* (AUC = 0.949), *HOXB5* (AUC = 0.908), *PTPN3* (AUC = 0.952), *MARCKSL1* (AUC = 0.962), *PTCH1* (AUC = 0.981), and *CDC20* (AUC = 0.956) could significantly distinguish BCC samples and healthy controls, implying that these genes might serve as diagnostic makers for BCC detection (Figure 5).

## 4. Discussion

In the present study, we performed an integrated bioinformatics analysis using five microarray datasets about BCC. In total, 804 DEGs (414 upregulated and 390 downregulated genes) were screened between BCC and normal control tissues. The GO functional analysis showed that several genes, including *CYFIP2*, *HOXB5*, *EGFR*, *FOXN3*, *PTPN3*, *CDC20*, *MARCKSL1*, *FAS*, and *PTCH1*, primarily played pivotal roles in cellular processes. Moreover, the expression patterns of six genes (*CYFIP2*, *HOXB5*, *FOXN3*, *PTPN3*, *MARCKSL1*, and *FAS*) were validated in an external noncoding RNA dataset. Additionally, *CYFIP2*, *HOXB5*, *PTPN3*, *MARCKSL*, *CDC20*, and *PTCH1* had superior diagnostic values for BCC prediction.

Overwhelming evidence has suggested that the patched gene (*PTCH*) family played vital roles in BCC induction and progression [19, 20]. A previous study highlighted that *PTCH* could encode a receptor for the hedgehog signaling pathway which was important for vertebrate development and tumorigenesis [21]. Undén et al. found that the expression level of *PTCH* mRNA was overexpressed in BCC cells compared with nontumor epidermal cells, which was similar to our finding that *PTCH1* was upregulated in BCC patients [22]. Interestingly, numerous researchers pointed out that approximately 90% of function mutations in *PTCH1* were strongly related to BCC initiation and progression [23]. Gianferante et al. evaluated 18 nevoid BCC syndrome National Cancer Institute families by whole exome sequencing and found that 89% of families exhibited a pathogenic *PTCH1* mutation [24]. A recent investigation has reported that *PTCH1* had two mutational statuses (germinal and somatic mutation). Moreover, there was a higher expression level of *PTCH1* in BCC with germinal and somatic *PTCH1* mutations than that only with germinal *PTCH1* mutation [25]. Additionally, our functional analyses showed that *PTCH1* played significant roles in cell proliferation and BCC pathway. However, few reports investigated the detailed molecular mechanisms of the biological roles of *PTCH1* on BCC cell proliferation and growth. Herein, we performed a ROC curve analysis of *PTCH1*, and the result indicated that this gene was capable of discriminating BCC patients and healthy controls with a relatively high AUC value (AUC = 0.981). Taken together, our findings suggested that *PTCH1* might be involved in the pathogenesis of BCC and has a great diagnostic value for BCC detection.

We found that four genes (upregulated *CYFIP2* and *MARCKSL1* and downregulated *HOXB5* and *PTPN3*) were also key players in cell biological processes. Furthermore, their expression patterns were validated by the differential expression analysis based on an external dataset. Besides, these four genes could clearly differentiate BCC patients from normal controls. *CYFIP2* was reported to be a direct *p53* target gene, and its overexpression was closely linked with the development of various cancers [26, 27]. Ling et al. analyzed *p53* mutations in sporadic and hereditary BCC tumors, and genetic alterations of *p53* were responsible for BCC progression [28]. Later on, Huang et al. stated that *p53* activation by imiquimod contributed to cell apoptosis of a skin BCC/KMC1 cell line [29]. These findings provided indirect evidence for the hypothesis that *CYFIP2* participated in the pathogenic mechanism of BCC. *HOXB5* is a member of the homeobox (*HOX*) gene B cluster and plays key roles in several cancers [30, 31]. Lee et al. suggested that *HOXB5* increased cell proliferation and invasiveness in estrogen receptor- (ER-) positive breast cancer [32]. More interestingly, this research group also found that *HOXB5* could upregulate *EGFR* expression and thereby promote *HOXB5*-driven invasion in ER-positive breast cancer [33]. In this work, we noted that *EGFR* was involved in cell cycle and proliferation and served as a pivotal hub gene in PPI analysis. Whether the *HOXB5/EGFR* axis participated in BCC development remains to be illuminated in the following analysis. *PTPN3* (also known as *PTPH1*) was also reported to be strongly correlated with diverse cancers including colorectal cancer and gastric adenocarcinoma [34–36]. Furthermore, Li et al. unraveled that the *PTPN3* depletion could block the degradation of *EGFR*, which accelerated cell proliferation and tumorigenicity in lung cancer cells [37]. The potential roles of the *PTPN3*/*EGFR* axis on BCC occurrence also need to be clarified in the future. *MARCKSL1* is a cytoskeletal regulator and implicated with the initiation of multiple cancers [38]. Our results implied that *MARCKSL1* might be a promising diagnostic maker for BCC prediction. However, the underlying pathogenic mechanisms of the four-gene signature (*CYFIP2*, *MARCKSL1*, *HOXB5*, and *PTPN3*) for BCC progression have not been expatiated.

The *FAS* gene contains a highly conserved cytoplasmic death domain and encodes *a* transmembrane protein of the tumor necrosis factor receptor superfamily. Existing evidence has shown that *FAS* could bind to a death ligand, *FAS* ligand (FASL), to induce the cell apoptotic pathway [39]. The early research indicated that *FAS* normally was underexpressed and even undetectable in BCC while there was a high *FASL* level, possibly inducing cell death and contributing to cancerization [40]. A later study examined the expression level of FAS/FASL in BCCs using the immunohistochemical method. The results implied that FASL was located on the cell membrane of keratinocytes at the basal cell layers and FAS/FASL was markedly decreased in BCC [41]. Wang et al. revealed that *FAS/FASL* mRNA expression and protein levels were reduced in the BCC compared to the normal skin samples and FASL immunostaining levels were strongly related to the ability of tumor invasiveness and metastasis [42]. Similarly, we found that there was a lower *FAS* expression in BCC tissues than healthy controls, which was also confirmed by a bioinformatics analysis with another BCC dataset. Therefore, we speculated that *FAS* might be a key gene driver in BCC development. The other two genes' (*CDC20* and *FOXN3*) levels were increased in BCC tissues compared to normal tissues, and functional analysis showed that they were mainly involved in the cell cycle process. We also observed that *CDC20* exhibited a good performance for BCC diagnosis based on a ROC analysis. Therefore, we inferred that the aberrant expression of *CDC20* and *FOXN3* was probably associated with BCC development. However, the influence of these two genes on BCC pathogenesis has not been fully investigated.

There are still limitations in our analysis. Firstly, only one external noncoding RNA dataset GSE74858 was obtained and used to evaluate expression levels of identified DEGs due to lack of gene expression profile of BCC. Notably, the expression patterns of six genes (*CYFIP2*, *HOXB5*, *FOXN3*, *PTPN3*, *MARCKSL1*, and *FAS*) were consistent with our initial differential expression analysis in the training dataset. However, the expression levels of other key genes still need to be further evaluated by using a larger sample size. Secondly, the corresponding experimental assays such as cell and animal experiments were not performed to validate our conclusion primarily due to lack of enough patients' samples and limited research funding. Thirdly, the underlying molecular mechanism between several signaling pathways involving key genes and BCC development remains to be deciphered. Fourthly, the relevant clinical characteristics are also required to be collected to assess BCC prognosis.

In summary, nine gene signatures (*CYFIP2*, *HOXB5*, *EGFR*, *FOXN3*, *PTPN3*, *CDC20*, *MARCKSL1*, *FAS*, and *PTCH1*) may play central roles in the initiation and progression of BCC, which provides deeper insights into BCC management. However, experimental verification and integrated bioinformatics analyses still need to be carried out in the future.

## Figures and Tables

**Table 1:** The microarray datasets for basal cell carcinoma.

GEO accession	Control	Case	Platform	Year	Country	Author
GSE103439	2	4	GPL570 [HG-U133_Plus_2] Affymetrix Human Genome U133 Plus 2.0 Array	2017	Japan	Yoshiaki Tabuchi
GSE53462	5	16	GPL10558 Illumina Human HT-12 V4.0 expression beadchip	2014	South Korea	Hyun Goo Woo
GSE42109	10	11	GPL570 [HG-U133_Plus_2] Affymetrix Human Genome U133 Plus 2.0 Array; GPL571 [HG-U133A_2] Affymetrix Human Genome U133A 2.0 Array	2013	USA	Mayte Suarez-Farinas
GSE39612	64	2	GPL570 [HG-U133_Plus_2] Affymetrix Human Genome U133 Plus 2.0 Array	2012	USA	Paul Harms
GSE7553	4	15	GPL570 [HG-U133_Plus_2] Affymetrix Human Genome U133 Plus 2.0 Array	2008	USA	Sean Yoder

GEO: Gene Expression Omnibus.

**Table 2:** The functional analyses of differentially expressed genes in BCC.

Category	Term	Count	*P* value	Genes
GOTERM_BP (top 5)	Cellular component organization	183	3.92*E* − 10	*RNH1*, *KDM1A*, *ZWILCH*, *DSCC1*, *HDAC11*, *INPPL1*, *TESK2*, *BUB1B*, *UBE3A*, *FNBP1L*,…
	Cellular process	507	1.22*E* − 09	*CYFIP2*, *RPL7*, *HOXA5*, *HOXB5*, *EGFR*, *FOXN3*, *PTPN3*, *CDC20*, *FAS*, *PTCH1*, *IDH2*,…
	Cell cycle phase	49	8.98*E* − 09	*ZWILCH*, *CDC20*, *EGFR*, *FOXN3*, *HSPA2*, *NDC80*, *ZWINT*, *TPX2*, *CENPF*, *KIF18A*, *MC1R*,…
	M phase	42	1.86*E* − 08	*CDCA3*, *ZWILCH*, *DSCC1*, *CDCA8*, *BUB1B*, *TTK*, *MKI67*, *NCAPH*, *CDC20*, *PSMC3IP*,…
	Cell proliferation	48	2.47*E* − 08	*ACHE*, *CSE1L*, *EGFR*, *PTCH1*, *WNT5A*, *EMP1*, *SOX11*, *CBFA2T3*, *EREG*, *TPX2*,…

GOTERM_CC (top 5)	Intracellular part	552	1.83*E* − 12	*CYFIP2*, *PTPN3*, *EGFR*, *CDC20*, *FAS*, *FOXN3*, *NUP107*, *RNH1*, *TESK2*, *NOC2L*,…
	Intracellular	560	5.23*E* − 11	*CYFIP2*, *PTPN3*, *EGFR*, *CDC20*, *FAS*, *FOXN3*, *SPI1*, *NUP107*, *RNH1*, *TESK2*,…
	Cytoplasm	403	4.48*E* − 09	*CYFIP2*, *PTPN3*, *EGFR*, *CDC20*, *FAS*, *RNH1*, *TESK2*, *FNBP1L*, *RPL7*, *AKT1*,…
	Intracellular organelle	473	4.91*E* − 09	*PTPN3*, *EGFR*, *CDC20*, *FAS*, *FOXN3*, *SPI1*, *NUP107*, *RNH1*, *TESK2*, *NOC2L*,…
	Spindle	26	5.26*E* − 09	*CDCA8*, *BUB1B*, *TTK*, *CDC14A*, *CDC20*, *CCNB1*, *RACGAP1*, *HAUS7*, *MYC*, *AKT1*,…

GOTERM_MF	Protein binding	448	2.09*E* − 12	*CYFIP2*, *EGFR*, *FOXN3*, *PTPN3*, *CDC20*,*FAS*, *PTCH1*, *MARCKSL1*, *CNTFR*, *SPI1*,…
	Binding	603	7.51*E* − 09	*CYFIP2*, *EGFR*, *FOXN3*, *PTPN3*, *CDC20*, *FAS*, *PTCH1*, *MARCKSL1*, *HOXB5*, *CNTFR*,…
	Ribonuclease H activity	4	1.21*E* − 04	*FEN1*, *RNASEH2A*, *RNH1*, *EXO1*

GO: Gene Ontology; BP: biological process; MF: molecular function; CC: cellular component; BCC: basal cell carcinoma.

## Data Availability

The datasets used and/or analyzed during the current study are available from the corresponding author on reasonable request.

## Supplementary Materials

Supplementary Materials The whole R sentences for data processing and identification of differentially expressed genes in this study.

## References

[1] Ballester-Sánchez R., Pons-Llanas O., Candela-Juan C. (2015). Efficacy and safety of electronic brachytherapy for superficial and nodular basal cell carcinoma. *Journal of Contemporary Brachytherapy*.

[2] Verkouteren J. A. C., Ramdas K. H. R., Wakkee M., Nijsten T. (2017). Epidemiology of basal cell carcinoma: scholarly review. *The British Journal of Dermatology*.

[3] Rubin A. I., Chen E. H., Ratner D. (2005). Basal-cell carcinoma. *The New England Journal of Medicine*.

[4] Chen Y., Liu J. (2019). The prognostic roles of cyclooxygenase-2 for patients with basal cell carcinoma. *Artificial Cells, Nanomedicine, and Biotechnology*.

[5] Flohil S. C., Seubring I., van Rossum M. M., Coebergh J. W. W., de Vries E., Nijsten T. (2013). Trends in basal cell carcinoma incidence rates: a 37-year Dutch observational study. *The Journal of Investigative Dermatology*.

[6] Tanese K. (2019). Diagnosis and management of basal cell carcinoma. *Current Treatment Options in Oncology*.

[7] Wadlow R., Ramaswamy S. (2005). DNA microarrays in clinical Cancer Research. *Current Molecular Medicine*.

[8] Fang M., Wee S. A., Ronski K., Fan H., Tao S., Lin Q. (2007). Evidence of EGR1 as a differentially expressed gene among proliferative skin diseases. *Genomic Medicine*.

[9] Heller E. R., Gor A., Wang D. (2013). Molecular signatures of basal cell carcinoma susceptibility and pathogenesis: a genomic approach. *International Journal of Oncology*.

[10] Yunoki T., Tabuchi Y., Hirano T., Miwa S., Imura J., Hayashi A. (2018). Gene networks in basal cell carcinoma of the eyelid, analyzed using gene expression profiling. *Oncology Letters*.

[11] Jee B. A., Lim H., Kwon S. M. (2015). Molecular classification of basal cell carcinoma of skin by gene expression profiling. *Molecular Carcinogenesis*.

[12] Barrett T., Troup D. B., Wilhite S. E. (2007). NCBI GEO: mining tens of millions of expression profiles--database and tools update. *Nucleic Acids Research*.

[13] Ramasamy A., Mondry A., Holmes C. C., Altman D. G. (2008). Key issues in conducting a meta-analysis of gene expression microarray datasets. *PLoS Medicine*.

[14] Marot G., Foulley J. L., Mayer C. D., Jaffrezic F. (2009). Moderated effect size and P-value combinations for microarray meta-analyses. *Bioinformatics*.

[15] Shannon P., Markiel A., Ozier O. (2003). cytoscape: a software environment for integrated models of biomolecular interaction networks. *Genome Research*.

[16] Xie C., Mao X., Huang J. (2011). KOBAS 2.0: a web server for annotation and identification of enriched pathways and diseases. *Nucleic Acids Research*.

[17] Tang Y., Li M., Wang J., Pan Y., Wu F. X. (2015). CytoNCA: a cytoscape plugin for centrality analysis and evaluation of protein interaction networks. *Bio Systems*.

[18] Liu Z. K., Zhang R. Y., Yong Y. L. (2019). Identification of crucial genes based on expression profiles of hepatocellular carcinomas by bioinformatics analysis. *PeerJ*.

[19] Fan H., Oro A. E., Scott M. P., Khavari P. A. (1997). Induction of basal cell carcinoma features in transgenic human skin expressing Sonic Hedgehog. *Nature Medicine*.

[20] Lacour J. P. (2002). Carcinogenesis of basal cell carcinomas: genetics and molecular mechanisms. *The British Journal of Dermatology*.

[21] Stone D. M., Hynes M., Armanini M. (1996). The tumour-suppressor gene patched encodes a candidate receptor for Sonic hedgehog. *Nature*.

[22] Undén A. B., Zaphiropoulos P. G., Bruce K., Toftgård R., Ståhle-Bäckdahl M. (1997). Human patched (PTCH) mRNA is overexpressed consistently in tumor cells of both familial and sporadic basal cell carcinoma. *Cancer Research*.

[23] Bresler S. C., Padwa B. L., Granter S. R. (2016). Nevoid basal cell carcinoma syndrome (Gorlin syndrome). *Head and Neck Pathology*.

[24] Gianferante D. M., Rotunno M., Dean M. (2018). Whole-exome sequencing of nevoid basal cell carcinoma syndrome families and review of Human Gene Mutation Database PTCH1 mutation data. *Molecular Genetics & Genomic Medicine*.

[25] Martinez M. F., Romano M., Martinez A. (2019). Nevoid basal cell carcinoma syndrome: PTCH1 mutation profile and expression of genes involved in the hedgehog pathway in Argentinian patients. *Cells*.

[26] Saller E., Tom E., Brunori M. (1999). Increased apoptosis induction by 121F mutant p53. *The EMBO Journal*.

[27] Jackson R. S., Cho Y.-J., Stein S., Liang P. (2007). CYFIP2, a direct p53 target, is leptomycin-B sensitive. *Cell Cycle*.

[28] Ling G., Ahmadian A., Persson Å. (2001). *PATCHED* and *p53* gene alterations in sporadic and hereditary basal cell cancer. *Oncogene*.

[29] Huang S. W., Chang S. H., Mu S. W. (2016). Imiquimod activates p53-dependent apoptosis in a human basal cell carcinoma cell line. *Journal of Dermatological Science*.

[30] Zhang B., Li N., Zhang H. (2018). Knockdown of homeobox B5 (HOXB5) inhibits cell proliferation, migration, and invasion in non-small cell lung cancer cells through inactivation of the Wnt/*β*-catenin pathway. *Oncology Research*.

[31] Xu N., Wu Y. P., Yin H. B., Xue X. Y., Gou X. (2018). Molecular network-based identification of competing endogenous RNAs and mRNA signatures that predict survival in prostate cancer. *Journal of Translational Medicine*.

[32] Lee J. Y., Hur H., Yun H. J. (2015). HOXB5 promotes the proliferation and invasion of breast cancer cells. *International Journal of Biological Sciences*.

[33] Lee J. Y., Kim J. M., Jeong D. S., Kim M. H. (2018). Transcriptional activation of EGFR by HOXB5 and its role in breast cancer cell invasion. *Biochemical and Biophysical Research Communications*.

[34] Han S., Williams S., Mustelin T. (2000). Cytoskeletal protein tyrosine phosphatase PTPH1 reduces T cell antigen receptor signaling. *European Journal of Immunology*.

[35] Wu C. W., Chen J. H., Kao H. L. (2006). PTPN3 and PTPN4 tyrosine phosphatase expression in human gastric adenocarcinoma. *Anticancer Research*.

[36] Wang Z., Shen D., Parsons D. W. (2004). Mutational analysis of the tyrosine phosphatome in colorectal cancers. *Science*.

[37] Li M. Y., Lai P. L., Chou Y. T. (2015). Protein tyrosine phosphatase PTPN3 inhibits lung cancer cell proliferation and migration by promoting EGFR endocytic degradation. *Oncogene*.

[38] Chen Z., Liu Y., Yao L., Guo S., Gao Y., Zhu P. (2018). The long noncoding RNA lncZic2 drives the self-renewal of liver tumor-initiating cells via the protein kinase C substrates MARCKS and MARCKSL1. *The Journal of Biological Chemistry*.

[39] Dicker T., Siller G., Saunders N. (2002). Molecular and cellular biology of basal cell carcinoma. *The Australasian Journal of Dermatology*.

[40] Gutierrez-Steil C., Wrone-Smith T., Sun X., Krueger J. G., Coven T., Nickoloff B. J. (1998). Sunlight-induced basal cell carcinoma tumor cells and ultraviolet-B-irradiated psoriatic plaques express Fas ligand (CD95L). *The Journal of Clinical Investigation*.

[41] Jang T. J. (2002). Expression of CD40 and Fas ligand in Bowen's disease, squamous cell carcinoma and basal cell carcinoma. *Yonsei Medical Journal*.

[42] Wang X. Y., Zhang R., Lian S. (2011). Aberrant expression of Fas and FasL pro-apoptotic proteins in basal cell and squamous cell carcinomas. *Clinical and Experimental Dermatology*.

