# Supplementary material for: Identification of Potential Biomarkers Associated with Basal Cell Carcinoma
Source: Biomed Res Int. 2020 Apr 17;2020:2073690. doi: 10.1155/2020/2073690 (PMC7189327; doi:10.1155/2020/2073690)
Supplement: Supplementary Materials — The whole R sentences for data processing and identification of differentially expressed genes in this study. [file 2073690.f1.docx]

The whole R sentences for data processing and identification of differentially expressed genes in this study are listed as follows.

1. data reading

df11<-read.csv(“GSE7553.csv”,row.names = 1)

df21<-read.csv(“GSE39612.csv”,row.names = 1)

df31<-read.csv(“GSE42109.csv”,row.names = 1)

df41<-read.csv(“GSE53462.csv”,row.names = 1)

df51<-read.csv(“GSE103439.csv”,row.names = 1)

1. sample data reading

smplbl<-read.csv(‘smp-data-7553.csv’,header=FALSE)

class1<-factor(x=t(smplbl),levels=c(0,1),labels=c(‘control’, ‘case’))

idx<-order(class1)

class11<-class1[idx]

smplbl<-read.csv(‘smp-data-39612.csv’,header=FALSE)

class2<-factor(x=t(smplbl),levels=c(0,1),labels=c(‘control’, ‘case’))

idx<-order(class2)

class21<-class2[idx]

smplbl<-read.csv(‘smp-data-42109.csv’,header=FALSE)

class3<-factor(x=t(smplbl),levels=c(0,1),labels=c(‘control’, ‘case’))

idx<-order(class3)

class31<-class3[idx]

smplbl<-read.csv(‘smp-data-53462.csv’,header=FALSE)

class4<-factor(x=t(smplbl),levels=c(0,1),labels=c(‘control’, ‘case’))

idx<-order(class4)

class41<-class4[idx]

smplbl<-read.csv(‘smp-data-103439.csv’,header=FALSE)

class5<-factor(x=t(smplbl),levels=c(0,1),labels=c(‘control’, ‘case’))

idx<-order(class5)

class51<-class5[idx]

1. data merging

data2test<-list(df11,df21,df31,df41,df51)

sampleclass<-list(class11,class21,class31,class41,class51)

1. Identification of differentially expressed genes

resES<-EScombination(esets=data2test,classes=sampleclass)

rawpvalES<-2*(1-pnorm(abs(resES$TestStatistic)))

fdrES<-p.adjust(rawpvalES, “BH”)

indES<-which(fdrES<0.05)

length(indES)

1. differentially expressed genes exporting

data<-cbind(df11,df21,df31,df41,df51)

statelabel<-c(as.numeric(class11),as.numeric(class21),as.numeric(class31),as.numeric(class41),as.numeric(class51))

controlidx<-which(statelabel==1)

caseidx<-which(statelabel==2)

updown<-sign(rowMeans(data[,caseidx],na.rm=TRUE)-rowMeans(data[,controlidx],na.rm=TRUE))

expCase<-rowMeans(data[,caseidx],na.rm=TRUE)

expCtrl<-rowMeans(data[,controlidx],na.rm=TRUE)

geneid<-row.names(data)

Result<-list(geneid[indES],expCase[indES],expCtrl[indES],rawpvalES[indES],fdrES[indES],updown[indES])

write.table(Result,file=“mRNA-result.txt”,sep=“\t”)
